# Sitafloxacin reduces tumor necrosis factor alpha (TNFα) converting enzyme (TACE) phosphorylation and activity to inhibit TNFα release from lipopolysaccharide-stimulated THP-1 cells

**DOI:** 10.1038/s41598-021-03511-5

**Published:** 2021-12-17

**Authors:** Ippei Sakamaki, Michika Fukushi, Wakana Ohashi, Yukie Tanaka, Kazuhiro Itoh, Kei Tomihara, Yoshihiro Yamamoto, Hiromichi Iwasaki

**Affiliations:** 1 Department of Infectious Diseases, Faculty of Medical Sciences, University of Fukui, 23-3, Matsuokashimoaizuki, Eiheiji-cho, Yoshida-gun, Fukui, 910-1193 Japan; 2 Department of Clinical Infectious Diseases, Toyama University Hospital, Toyama, Japan; 3 Department of Diagnostic Pathology, Graduate School of Medicine and Pharmaceutical Sciences, University of Toyama, Toyama, Japan; 4 Division of Biochemistry, Faculty of Pharmacy and Graduate School of Pharmaceutical Science, Keio University, Tokyo, Japan; 5 Department of Integrative Vascular Biology, Faculty of Medical Sciences, University of Fukui, Fukui, Japan; 6 Division of Oral and Maxillofacial Surgery, Niigata University Graduate School of Medical and Dental Sciences, Niigata, Japan; 7 Department of Infection Control and Prevention, University of Fukui Hospital, Fukui, Japan

**Keywords:** Immunology, Microbiology

## Abstract

Sepsis is a systemic reaction to an infection and resulting in excessive production of inflammatory cytokines and chemokines. It sometimes results in septic shock. The present study aimed to identify quinolone antibiotics that can reduce tumor necrosis factor alpha (TNFα) production and to elucidate mechanisms underlying inhibition of TNFα production. We identified quinolone antibiotics reduced TNFα production in lipopolysaccharide (LPS)-stimulated THP-1 cells. Sitafloxacin (STFX) is a broad-spectrum antibiotic of the quinolone class. STFX effectively suppressed TNFα production in LPS-stimulated THP-1 cells in a dose-dependent manner and increased extracellular signal-regulated kinase (ERK) phosphorylation. The percentage of intracellular TNFα increased in LPS-stimulated cells with STFX compared with that in LPS-stimulated cells. TNFα converting enzyme (TACE) released TNFα from the cells, and STFX suppressed TACE phosphorylation and activity. To conclude, one of the mechanisms underlying inhibition of TNFα production in LPS-stimulated THP-1 cells treated with STFX is the inhibition of TNFα release from cells via the suppression of TACE phosphorylation and activity. STFX may kill bacteria and suppress inflammation. Therefore, it can be effective for sepsis treatment.

## Introduction

Sepsis is defined as life-threatening organ dysfunction caused by a dysregulated host response to infection^1^. Sepsis was first defined as sepsis-1 in 1991 and was redefined as sepsis-3 in 2016. Sepsis was further defined as a systemic inflammatory response syndrome caused by infection^2^. Particularly during gram-negative bacterial infection, lipopolysaccharide (LPS) stimulates cells to produce inflammatory cytokines and chemokines, which can sometimes result in septic shock. Inflammatory cytokines lower the blood pressure via blood vessels dilation and blood clotting within the capillaries of organs. These effects can aid the immune system in fighting infection, but can also be harmful. Thus, drugs that are not only effective against bacterial infections but also reduce inflammatory cytokines are required to avoid such harmful effects.

Treatment with such drugs may help prevent septic shock and reduce mortality. Some antibiotics such as tetracycline^3,4^, macrolide^5–7^ and oxazolidinone^8,9^ have effectively reduced the production of inflammatory cytokines.

Quinolones such as garenoxacin or moxifloxacin have also been reported to reduce inflammatory cytokines^10,11^.

Sitafloxacin (STFX) is a broad-spectrum antimicrobial agent^12^. STFX is effective against pneumococcal infections, and incidence of drug-resistant mutants is low in vitro conditions^13^. STFX was effective against *Haemophilus influenzae* pneumonia in a murine model^14^.

In a clinical study, STFX was also proven effective and safe in elderly patients with pneumonia including aspiration pneumonia in nursing homes^15^. STFX treatment was effective in patients with both acute complicated urinary tract infection and pyelonephritis caused by *Escherichia coli* producing extended-spectrum beta-lactamase (ESBL)^16^. Another study also reported that STFX was effective against the *E. coli* producing ESBL following 3 days of carbapenem therapy^17^.

STFX, a broad-spectrum oral fluoroquinolone, has been approved in Japan for the treatment of respiratory and urinary tract infections. However, whether STFX can be used for treating patients with sepsis or whether it suppresses the production of inflammatory cytokines and chemokines is unknown, which we aimed to determine in the present study.

## Results

### Tumor necrosis factor alpha (TNFα concentration was high in supernatants of 4 h LPS-stimulated THP-1 cells

**Figure 1:**
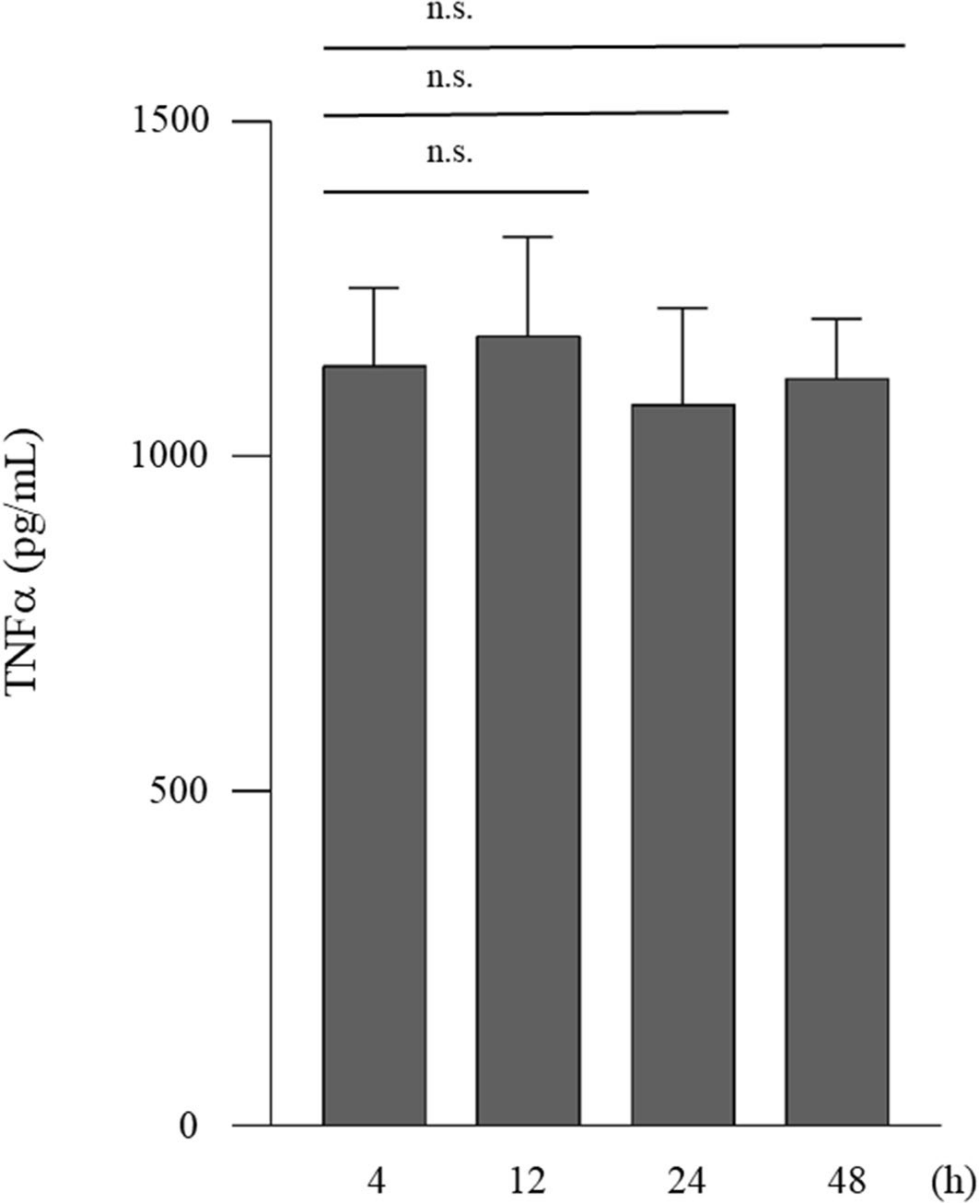
TNFα concentration did not change after 4 h. THP-1 cells (2 × 10^5^/mL) were stimulated by LPS (0.1 µg/mL) for 4 h, 12 h, 24 h or 48 h. Data are presented as the mean ± SD of 6 independent experiments.

TNFα concentration in the supernatants of THP-1 cells stimulated by LPS for 4 h, 12 h, 24 h or 48 h was 1135.21 ± 116.24 pg/mL, 1180.39 ± 148.17 pg/mL, 1078.65 ± 143.12 pg/mL, or 1116.81 ± 89.16 pg/mL, respectively (Fig. 1). TNFα concentration at 4 h was not significantly lower than that at 12 h, 24 h, or 48 h.

### STFX inhibited TNFα production significantly compared with other quinolones

**Figure 2:**
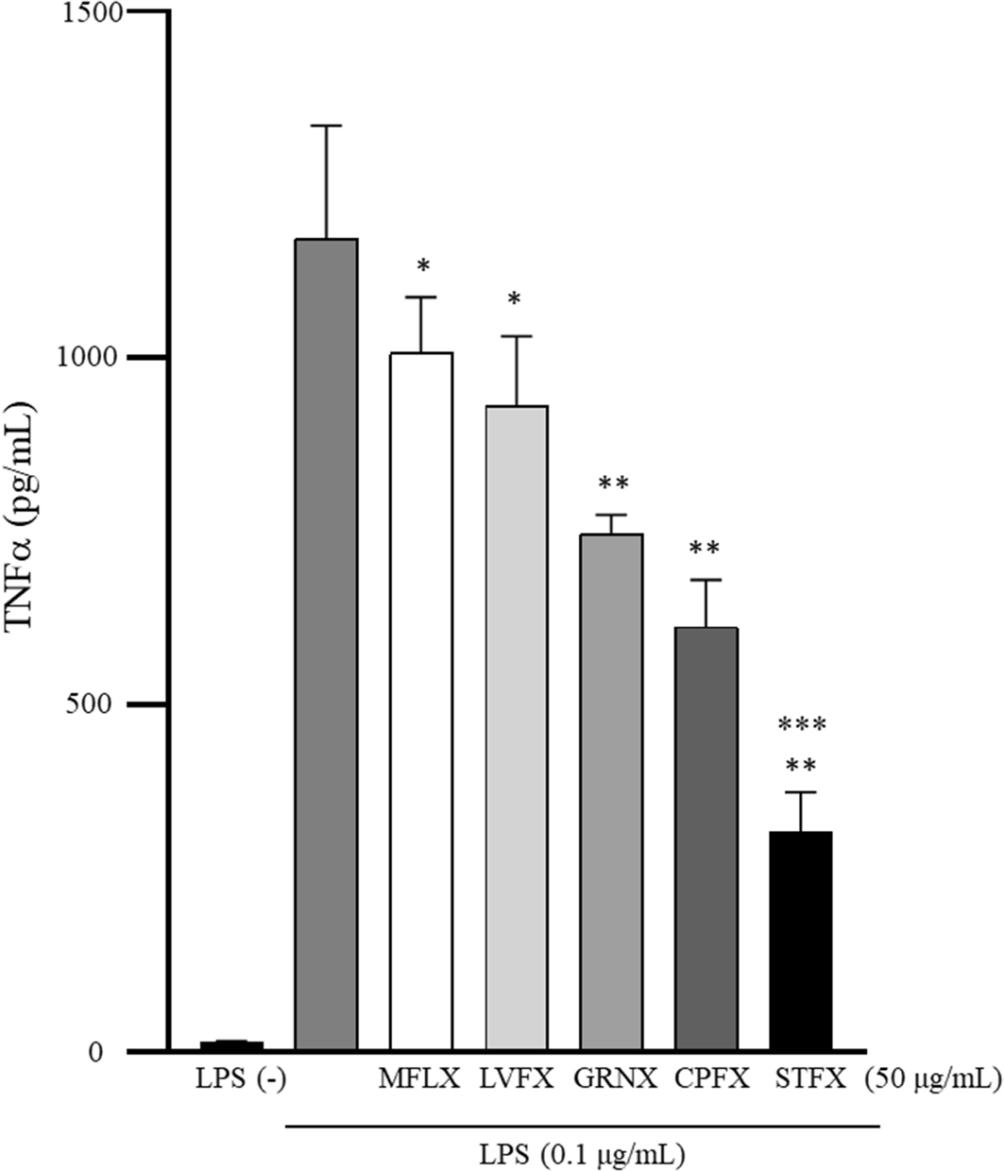
Sitafloxacin significantly reduced TNFα production. THP-1 cells (2 × 10^5^/mL) were stimulated by LPS (0.1 µg/mL) with several different quinolone antibiotics (50 µg/mL) for 4 h. Data are presented as mean ± SD of 6 independent experiments. *p < 0.05 vs. LPS alone. **p < 0.01 vs. LPS alone. ***p < 0.01 vs. MFLX, LVFX, GRNX, or CPFX. *LPS* lipopolysaccharide, *MFLX* moxifloxacin, *LVFX* levofloxacin, *GRNX* garenoxacin, *CPFX* ciprofloxacin, *STFX* sitafloxacin.

We examined which quinolones can inhibit TNFα production by determining of TNFα concentration in the supernatant of LPS-stimulated THP-1 cells treated with 50 µg/mL quinolone antibiotics. TNFα concentrations in these supernatants after moxifloxacin (MFLX), levofloxacin (LVFX), garenoxacin (GRNX), ciprofloxacin (CPFX) and STFX treatment were 1007.81 ± 79.92 pg/mL, 932.73 ± 99.14 pg/mL, 747.19 ± 27.76 pg/mL, 613.90 ± 67.56 pg/mL, or 316.90 ± 57.69 pg/mL, respectively. MFLX and LVFX treatments significantly reduced TNFα concentration than LPS treatment alone did (1173.49 ± 162.51 pg/mL) (p < 0.05). GRNX, CPFX, and STFX treatments significantly reduced TNFα concentrations than LPS alone treatment did (control) (p < 0.01). TNFα concentrations following LPS and STFX treatments were significantly lower than those following LPS and MFLX, LVFX, GRNX, or CPFX (p < 0.01), and STFX reduced TNFα concentration the most (Fig. 2).

### STFX inhibited TNFα production in a dose-dependent manner

**Figure 3:**
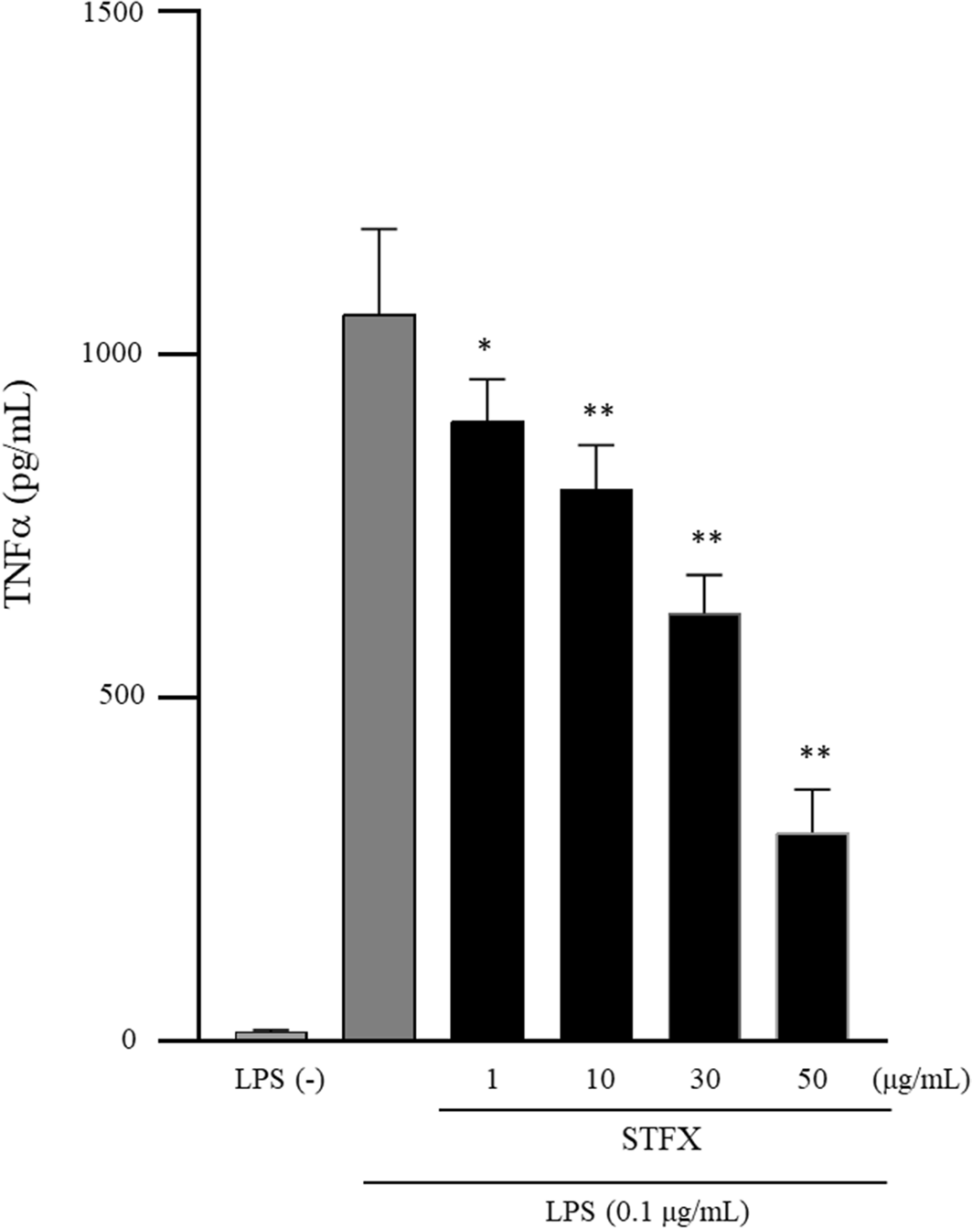
Sitafloxacin reduced TNFα in a dose-dependent manner. THP-1 cells (2 × 10^5^/mL) were stimulated by LPS (0.1 µg/mL) in the presence of various concentrations of STFX (1, 10, 30, and 50 µg/mL) for 4 h. Data are presented as the mean ± SD of 6 independent experiments. *p < 0.05, **p < 0.01 vs. LPS alone. *STFX* sitafloxacin.

Concentrations of TNFα in the supernatants of only LPS-stimulated THP-1 cells was 1057.80 ± 125.80 pg/mL Concentrations of TNFα in the supernatants of LPS-stimulated THP-1 cells in the presence of 1, 10, 30, and 50 µg/mL STFX were 903.26 ± 61.56 pg/mL (p < 0.05 vs. LPS alone), 803.20 ± 64.52 pg/mL (p < 0.01 vs. LPS alone), 622.61 ± 56.64 pg/mL (p < 0.01 vs. LPS alone), and 303.92 ± 63.42 pg/mL (p < 0.01 vs. LPS alone), respectively (Fig. 3).

### STFX inhibited the production of chemokines

**Figure 4:**
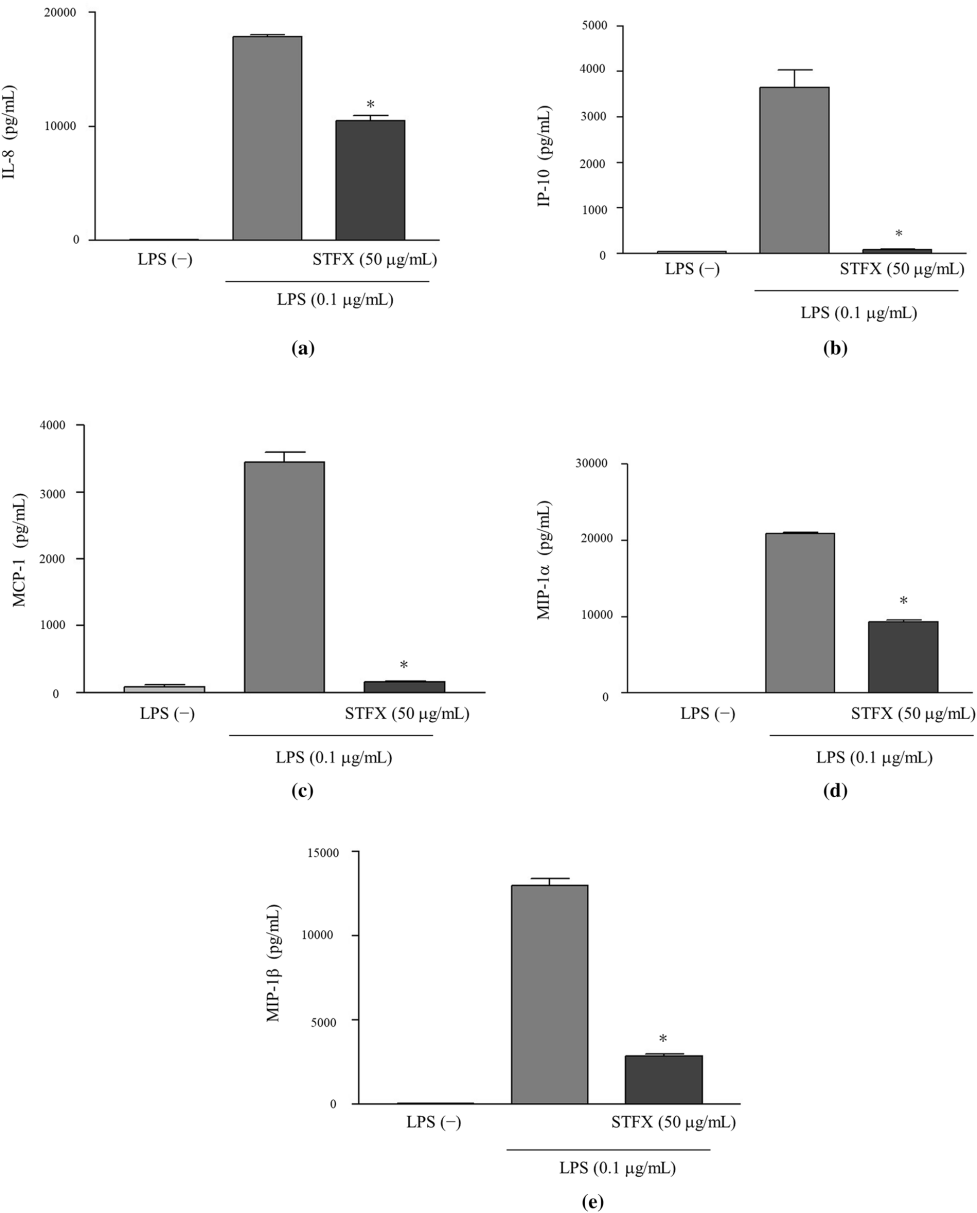
STFX reduced the levels of inflammatory chemokines. THP-1 cells (2 × 10^5^/mL) were stimulated by LPS (0.1 µg/mL) with STFX (50 µg/mL) for 4 h. Concentrations of IL-8 (**a**), IP-10 (**b**), MCP-1 (**c**), MIP-1α (**d**) and MIP-1β (**e**) were measured via multiplex bead immunoassays. Data are presented as the mean ± SD of 3 independent experiments. *p < 0.01 vs. LPS alone. *IL-8* interleukin-8, *IP-10* interferon inducible protein, *MCP-1* monocyte chemoattractant protein-1, *MIP-1α* macrophage inflammatory protein-1α, *MIP-1β* macrophage inflammatory protein-1β.

STFX inhibited not only TNFα production but also chemokines production, as indicated by additional experiments with LPS-stimulated THP-1 cells. The concentration of interleukin-8 (IL-8) in the supernatants of cells treated with 50 µg/mL STFX was significantly decreased to 10,472.00 ± 474.67 pg/mL compared with that of LPS alone (17,802.33 ± 190.07 pg/mL) (p < 0.01) (Fig. 4a). The concentrations of interferon inducible protein (IP-10) in the supernatants of cells treated with 50 µg/mL STFX was significantly decreased to 77.83 ± 9.70 pg/mL compared with that of the cells treated with LPS alone (3649.00 ± 377.59 pg/mL) (p < 0.01) (Fig. 4b). The concentration of monocyte chemoattractant protein-1 (MCP-1) in cell supernatants in the presence of 50 µg/mL STFX was also significantly decreased to 161.67 ± 11.59 pg/mL compared with that of LPS alone (3453.00 ± 148.55 pg/mL) (p < 0.01) (Fig. 4c). Furthermore, macrophage inflammatory protein-1α (MIP-1α concentrations in the supernatants of cells followed by treatment with 50 µg/mL STFX were significantly decreased to 9336.67 ± 206.50 pg/mL compared with that of the cells treated with LPS alone (20,859.33 ± 196.41 p/mL) (p < 0.01) (Fig. 4d). The supernatant concentration of macrophage inflammatory protein-1β (MIP-1β from the cells treated with 50 µg/mL STFX was also significantly decreased to 2844.67 ± 135.43 pg/mL compared with that of the cells treated with LPS alone (12,950.67 ± 409.62 pg/mL) (p < 0.01) (Fig. 4e).

### The phosphorylated form of extracellular signal-regulated kinase (ERK) increased treated with STFX

**Figure 5:**
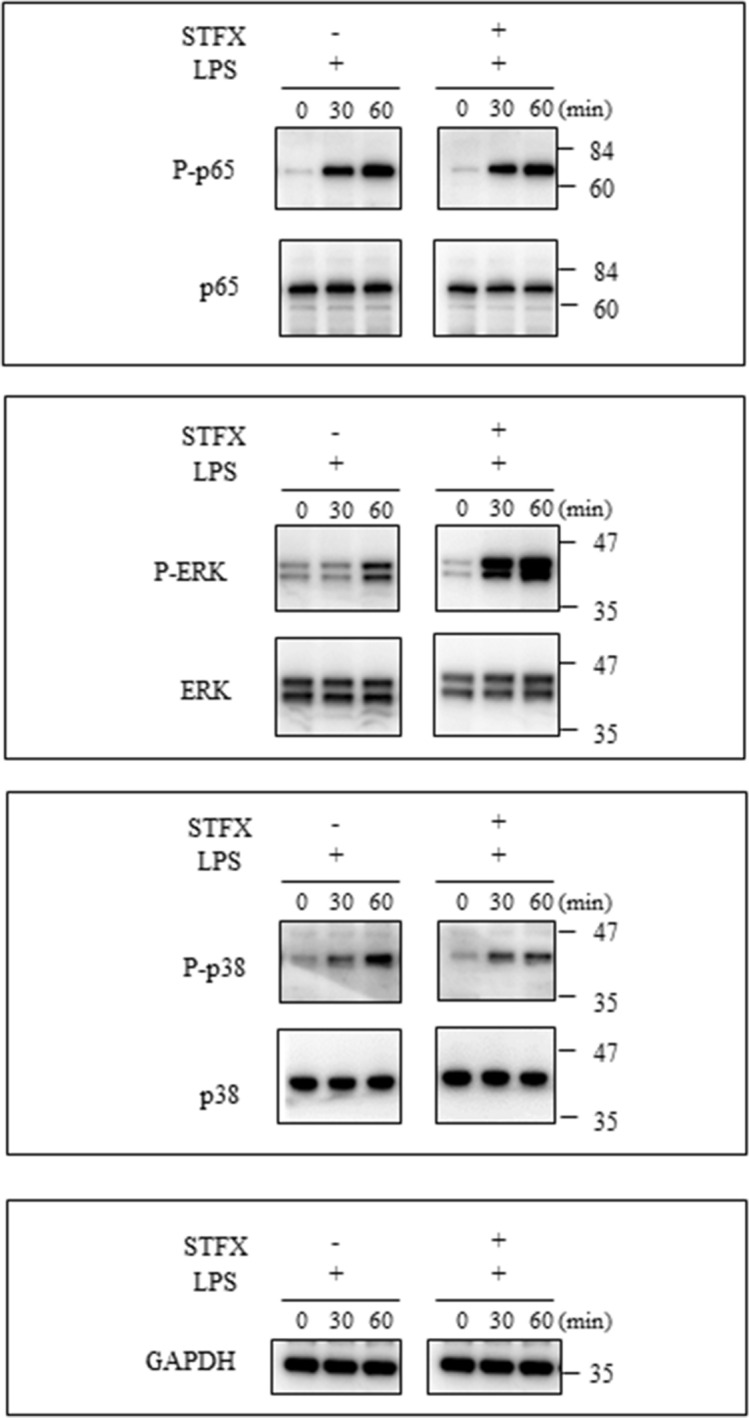
STFX did not inhibit signaling of TNFα production. THP-1 cells (2 × 10^5^/mL) were stimulated by LPS (0.1 µg/mL) with or without STFX (50 µg/mL) for 30 min or 60 min. NF-κB, ERK and p38, and the phosphorylation of NF-κB, ERK and p38 were evaluated by western blotting. The data are representative of 3 independent experiments. *NF-κB* Nuclear factor-kappa B, *ERK* extracellular signal-regulated kinase.

THP-1 cells (2 × 10^5^/mL) were stimulated with LPS (0.1 µg/mL) with or without the presence of STFX (50 µg/mL) for 30 min and 60 min. The phosphorylated form of ERK increased after treatment with STFX and LPS compared with treatment with LPS alone. The phosphorylated forms of nuclear factor kappa B (NF-κB) and p38 did not decrease in the cells treated with STFX and LPS compared with those treated with LPS alone (Fig. 5). Supplementary Fig. S1 presents the full-length blot and image (online).

### STFX inhibited TNFα release from cells

**Figure 6:**
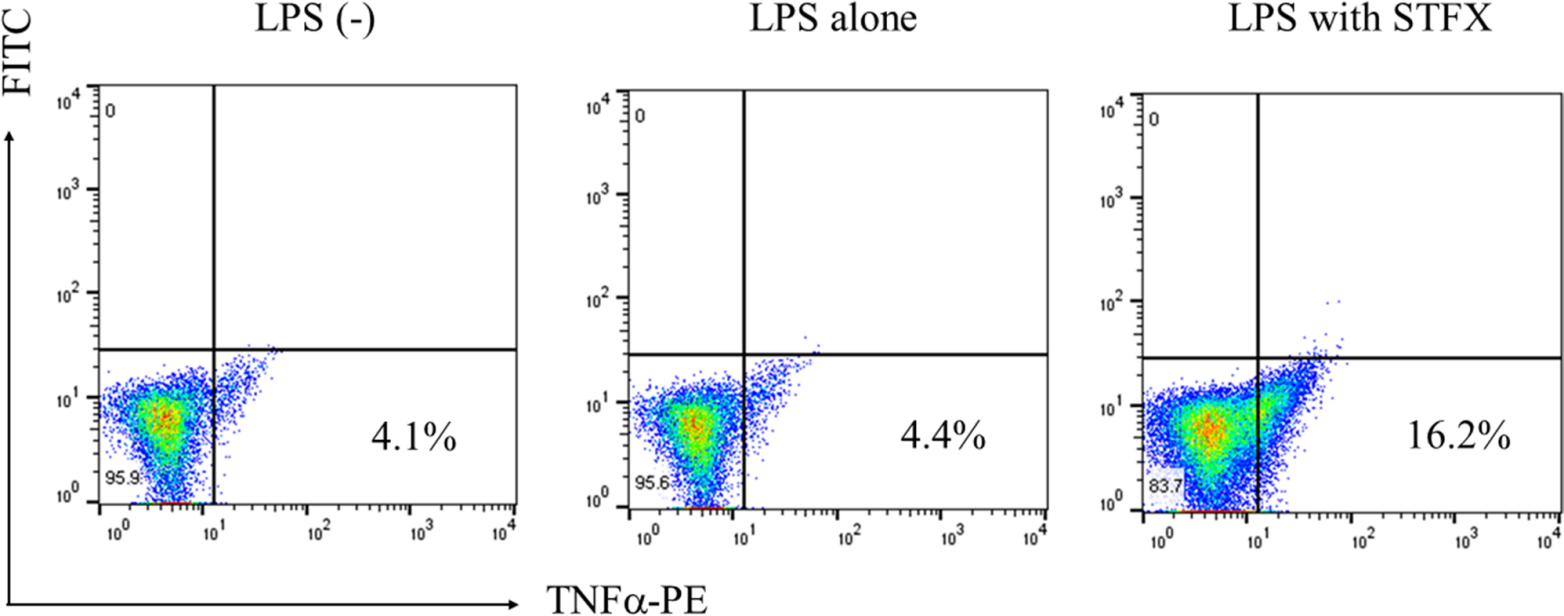
Intracellular TNFα levels increased with STFX**.** THP-1 cells (2 × 10^5^/mL) were stimulated by LPS (0.1 µg/mL) with or without STFX (50 µg/mL). After 4 h incubation, intracellular TNFα was stained with anti-TNFα antibody PE. The percentage of intracellular TNFα in LPS-stimulated cells in the presence or absence of STFX was evaluated by flow cytometry. The data are representative of 3 independent experiments.

THP-1 cells (2 × 10^5^/mL) were stimulated by LPS (0.1 µg/mL) with or without STFX (50 µg/mL). After 4 h of incubation, intracellular TNFα was stained with anti-TNFα antibody PE. The percentage of intracellular TNFα in the cells treated with STFX and LPS increased from 4.4 to 16.2% compared with that of the cells treated with LPS alone (Fig. 6).

### STFX reduced phosphorylation of TNFα converting enzyme (TACE) and TACE activity

**Figure 7:**
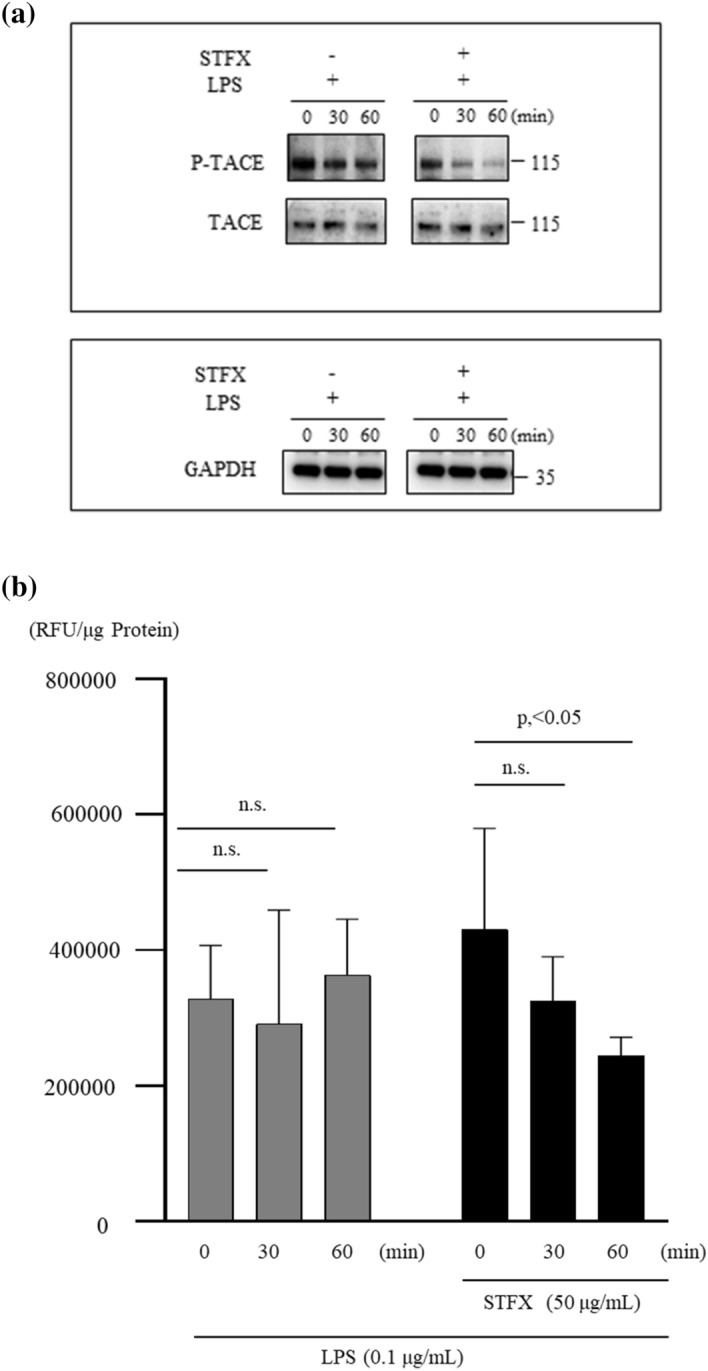
STFX reduced TACE phosphorylation and activity. THP-1 cells (2 × 10^5^/mL) were stimulated by LPS (0.1 µg/mL) with or without STFX (50 µg/mL) for 30 min or 60 min. (**a**) TACE or the phosphorylation of TACE was evaluated by western blotting. The data are representative of 3 independent experiments. (**b**) THP-1 cells (2 × 10^5^/mL) were stimulated by LPS (0.1 µg/mL) with or without STFX (50 µg/mL) for 30 min or 60 min. TACE activity was evaluated by ELISA. Data are presented the mean ± SD of 6 independent experiments.

THP-1 cells (2 × 10^5^/mL) were stimulated by LPS (0.1 µg/mL) with or without STFX (50 µg/mL) for 30 and 60 min. The phosphorylated form of TACE decreased after STFX and LPS treatment compared with LPS treatment alone. (Fig. 7a). Supplementary Fig. S2 presents the full-length blot and image (online). TACE activity of the cells treated for 60 min with STFX and LPS (244,805.70 ± 27,083.11 RFU/mg Protein) significantly decreased (p < 0.05) compared to TACE activity for 0 min (430,018.30 ± 149,978.40 RFU/mg protein) (Fig. 7b).

## Discussion

TNFα plays an important role in sepsis. TNFα blocking protected mice from sepsis symptoms^18^. Some clinical studies investigating the monoclonal antibodies produced against TNFα in patients with sepsis or septic shock have been reported^19–21^. The modulation of TNFα and other inflammatory cytokines and chemokines is considered important in the treatment of severe infectious diseases, especially sepsis or septic shock.

In the present study, TNFα concentration in the supernatant of LPS-stimulated THP-1 cells for 4 h was not significantly different from that of the cells treated for 12 h, 24 h, or 48 h. Some researchers have cultured THP-1 cells with LPS for 4 h and assessed TNFα concentration in the supernatant^4,22^. The authors of these papers revealed that TNFα level reached a maximum for 4 h incubation^4,22^. Therefore, we performed concentration experiments after 4 h of incubation. The concentration of TNFα in the supernatant at 4 h was a result of what happened in the cells earlier. Hence, we evaluated signaling pathway and TACE activity in the cells at 30 and 60 min.

STFX significantly reduced the concentration of TNFα in the supernatants of LPS-stimulated THP-1 cells than other quinolone antibiotics did; STFX also reduced the levels of IL-8, IP-10, MCP-1, MIP-1α and MIP-1β.

Some types of antibiotics can modulate inflammatory cytokines, but the mechanisms of cytokine inhibition may vary. A study has reported that minocycline inhibits IκB kinase α/β phosphorylation of NF-κB pathway in THP-1 cells^4^. Another study has reported that clarithromycin attenuates STAT6 phosphorylation^5^. Other studies have reported that macrolide antibiotics inhibited ERK and NF-κB signaling pathways^6,7^. GRNX and MFLX inhibited these signaling pathways to suppress the production of inflammatory cytokines. GRNX significantly inhibited the transcription and secretion of IL-8 induced by LPS-stimulated THP-1 cells by inhibiting ERK1/2 phosphorylation^10^. Furthermore, MFLX inhibited ERK1/2, JNK, and NF-κB activation in the cystic fibrosis epithelial cell line^11^.

Even when using similar quinolone antibacterial drugs, the mechanism of cytokine suppression differs depending on the characteristics of each drug. Previous studies have reported that quinolones with a cyclopropyl group at the N1 position and/or a piperazinyl group at the C7 position, can regulate inflammatory responses^23–25^. STFX consists of a fluorocyclopropene at the 1-position of the quinolone skeleton, a chlorine group at the 8-position, a spiroheptane group at the 7-position, and a quinolone with a chlorine group introduced at the 8-position. Such characteristics may cause differences in the spectrum of antibacterial activity and may also cause differences in anti-inflammatory effects.

In the present study, STFX suppressed TNFα production more strongly than the other quinolone antibiotics. It did not suppress the signaling pathways that produced TNFα but increased phosphorylated ERK. Flow cytometry analysis suggested that STFX inhibited the extracellular release of TNFα. TACE specifically cleaves pro-TNFα to release TNFα from cells^26,27^. Our study revealed that STFX reduced the phosphorylation and activity of TACE. One of the mechanisms inhibiting TNFα production by STFX might be interference with TNFα release from cells via the inhibition of TACE activity and phosphorylation but not the inhibition of signaling pathways.

STFX may be an effective drug for patients with bacterial infections because of its antimicrobial action and the simultaneous reduction of TNFα. STFX has been approved as an oral antibacterial drug and can be used to treat patients with sepsis or septic shock.

## Methods

### Reagents

Roswell Park Memorial Institute (RPMI) 1640 medium and fetal bovine serum (FBS) were purchased from Sigma-Aldrich (St. Louis, MO, USA). MFLX, GRNX and CPFX were purchased from FUJIFILM Wako Pure Chemical Corporation (Osaka, Japan). LVFX and STFX were provided by Daiichi Sankyo Company Limited. These antibiotics were diluted with RPMI 1640 at a concentration of 1.0 mg/mL to use as stock solutions. LPS from *Pseudomonas aeruginosa* serotype 10 (Sigma-Aldrich) was used to induce inflammatory responses. LPS was dissolved in RPMI 1640 medium at a concentration of 1.0 mg/mL and stored at – 80 °C until use.

### Cell culture and exposures

The human monocyte THP-1 cell line was purchased from the RIKEN Cell Bank (Ibaragi, Japan). The cells were cultured in RPMI 1640 medium supplemented with 10% FBS at 37 °C in humidified air with 5% CO_2_ and only exponentially growing cells were used for experiments. THP-1 cells (2 × 10^5^ cells/mL) were cultured with 0.1 µg/mL of LPS for 4 h, 12 h, 24 h, or 48 h. Data are presented as the mean ± standard deviation (SD) of 6 independent experiments.

THP-1 cells (2 × 10^5^ cells/mL) were cultured with LPS (0.1 µg/mL) in the presence or absence of antibiotics (MFLX, LVFX, GRNX, CPFX, and STFX) for 4 h. Following the incubation, supernatants were collected via centrifugation at 1500 rpm for 2 min at room temperature and stored at − 80 °C until further analysis. Data are presented as the mean ± SD of 6 independent experiments.

### ELISA

ELISA was performed using TNFα Human ELISA Kit (Invitrogen, Carlsbad, CA, USA) to determine TNFα concentration. The samples were read using an automated plate reader (Multiskan Spectrum; Thermo Scientific, Waltham MA, USA). Data are expressed as the mean ± SD of 6 independent experiments.

### Multiplex bead immunoassays

Multiplex bead immunoassays (Bio-Plex Suspension Array System, BIO-RAD Laboratories, Inc., CA, USA), which incorporate novel technology with color-coded beads and permits the simultaneous detection of up to 100 cytokines and chemokines in a single well of a 96-well microplate, was used for the simultaneous quantification of the following chemokines: IL-8, IP-10, MCP-1, MIP-1α and MIP-1β. Data expressed the mean ± SD of 3 independent experiments.

### Western blot analysis

Total protein was extracted from LPS-stimulated cells treated with antibiotics by using 200 µL of radioimmunoprecipitation assay buffer (FUJIFILM Wako, Osaka, Japan) containing a protease inhibitor cocktail (Nakalai Tesque, Kyoto, Japan) and the lysates were clarified by centrifugation (15,000 rpm, 10 min, 4 °C). Protein concentration was determined using Pierce 660 nm Protein Assay Kit (Thermo Scientific, Rockford, USA). Samples containing 10 µg of protein were run on a 10% polyacrylamide gel and electrotransferred onto a membrane filter (Immobilon-P; Millipore, Bedford, MA, USA). The membrane was blocked Blocking One (Nacalai Tesque) for 30 min, followed by incubation at room temperature for 1 h with a rabbit polyclonal antibody (Cell Signaling, Danvers, MA, USA) phospho-NF-κB p65, NF-κB p65, phospho-ERK, ERK, phospho-p38, p38, TACE and phospho-TACE (Abcam, Cambridge, UK). The membrane was then incubated at room temperature for 30 min with horseradish peroxidase-conjugated anti-mouse or anti-rabbit immunoglobulin G antibodies (GE Healthcare Bio-Science, Little Chalfont, England). Immunoreactive bands were visualized using enhanced chemiluminescence ImmunoStar LD (FUJIFILM Wako) and a FUSION-SOLO.7S.EDGE Chemilluminescence Imaging System (Vilber-Lourmat, 24 rue de Lamirault, 77090 Collégien, France). The data shown are representative of 3 independent experiments.

## Flow cytometry analysis of intracellular TNFα staining

THP-1 cells (2 × 10^5^/mL) were stimulated by LPS (0.1 µg/mL) with or without STFX (50 µg/mL) for 4 h. After incubation, the cells were fixed and permeabilized using a Fixation/Permeabilization Solution Kit (BD Biosciences, San Jose, CA, USA) according to the manufacturer’s protocol. Intracellular TNFα was stained using anti-TNFα antibody PE (BD Biosciences) for 1 h. The cells were washed and resuspended in phosphate-buffered saline (PBS) supplemented with 2% FBS and 0.05% NaN_3_. Intracellular TNFα was evaluated using FACS Canto II (BD Biosciences). Data shown are representative of 3 independent experiments.

### TACE activity

TACE activity was measured using SensoLyte 520 TACE (α-Secretase) Activity Assay Kit (Anaspec, Inc. CA, USA) according to manufacturer’s protocol. THP-1 cells (2 × 10^5^/mL) stimulated using LPS with or without STFX were washed with PBS. Assay buffer containing 0.1% Triton-X 100 was added to the cells or cell pellets. The cell suspension was collected in a microcentrifuge tube. The cell suspension was incubated at 4 °C for 10 min and then centrifuged for 10 min at 2500×*g*, at 4 °C. The supernatant was collected and stored at − 80 °C until use. Data are presented as the mean ± SD of 6 independent experiments.

### Statistical analysis

The values are expressed as the mean ± SD. Data were analyzed by Student’s t-test using a statistical software (Microsoft Excel 2008; Microsoft Corporation, Redmond, WA, USA), in which a p-value < 0.05 was considered statistically significant.

## Supplementary Information

Supplementary Figure S1.

Supplementary Figure S2.

## Data Availability

All data generated or analyzed during this study are included in this article.

## Abbreviations

CPFX: Ciprofloxacin
ESBL: Extended-spectrum beta-lactamase
ERK: Extracellular signal-regulated kinase
GRNX: Garenoxacin
IL-8: Interleukin-8
IP-10: Interferon inducible protein
LPS: Lipopolysaccharide
LVFX: Levofloxacin
MCP-1: Monocyte chemoattractant protein-1
MIP-1α: Macrophage inflammatory protein-1α
MIP-1β: Macrophage inflammatory protein-1β
MFLX: Moxifloxacin
NF-κB: Nuclear factor-kappa B
RIPA: Radioimmunoprecipitation assay
SIRS: Systemic inflammatory response syndrome
STFX: Sitafloxacin
TACE: Tumor necrosis factor α converting enzyme
TNFα: Tumor necrosis factor alfa
