# Supplementary figures and images for: Sitafloxacin reduces tumor necrosis factor alpha (TNFα) converting enzyme (TACE) phosphorylation and activity to inhibit TNFα release from lipopolysaccharide-stimulated THP-1 cells
Source: Sci Rep. 2021 Dec 17;11:24154. doi: 10.1038/s41598-021-03511-5 (PMC8683466; doi:10.1038/s41598-021-03511-5)

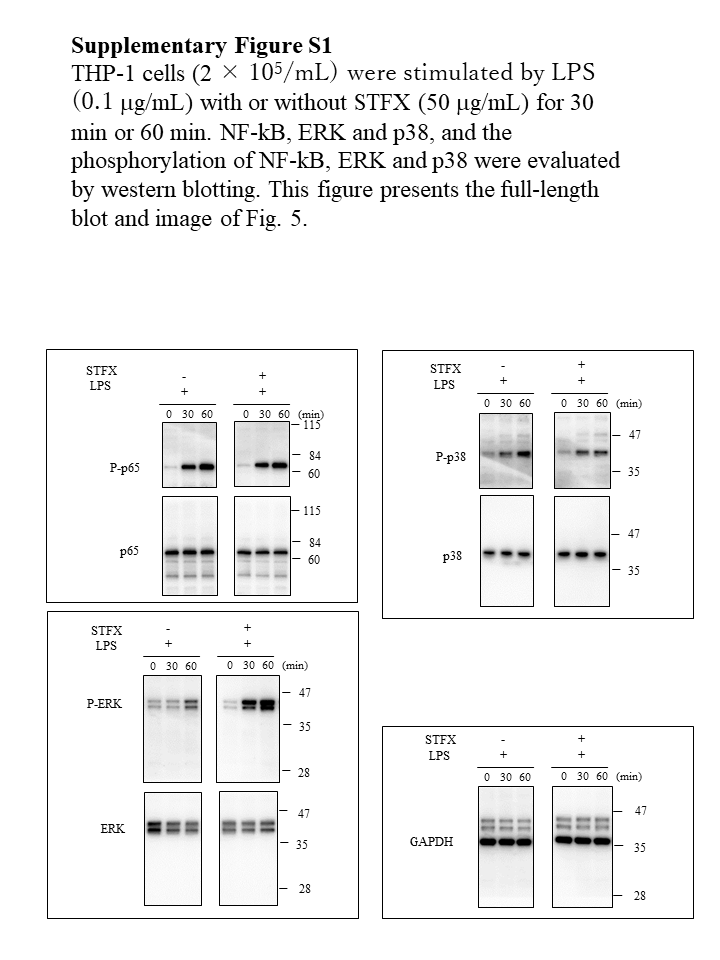

Supplement: Supplementary file 1 — Supplementary Figure S1. [file 41598_2021_3511_MOESM1_ESM.tif]

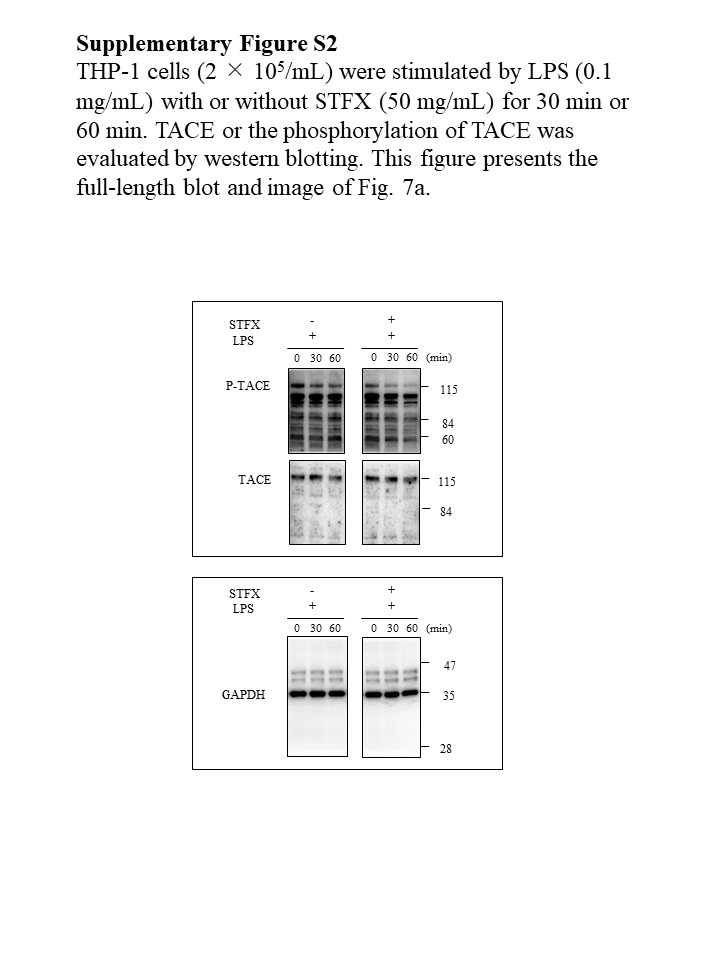

Supplement: Supplementary file 2 — Supplementary Figure S2. [file 41598_2021_3511_MOESM2_ESM.tif]
